# Structure of the cyanobactin oxidase ThcOx from *Cyanothece* sp. PCC 7425, the first structure to be solved at Diamond Light Source beamline I23 by means of S-SAD

**DOI:** 10.1107/S2059798316015850

**Published:** 2016-10-28

**Authors:** Andrew F. Bent, Greg Mann, Wael E. Houssen, Vitaliy Mykhaylyk, Ramona Duman, Louise Thomas, Marcel Jaspars, Armin Wagner, James H. Naismith

**Affiliations:** a BSRC, University of St Andrews, North Haugh, St Andrews, Fife KY16 9ST, Scotland; b Marine Biodiscovery Center, Department of Chemistry, University of Aberdeen, Meston Walk, Aberdeen AB24 3UE, Scotland; c Pharmacognosy Department, Faculty of Pharmacy, Mansoura University, Mansoura 35516, Egypt; d Diamond Light Source Ltd, Diamond House, Harwell Science and Innovation Campus, Didcot OX11 0DE, England; e State Key Laboratory of Biotherapy and Collaborative Innovation Center for Biotherapy, West China Hospital, Sichuan University, Chengdu, People’s Republic of China

**Keywords:** cyanobactins, azoline oxidase, S-SAD, RIPPs, phasing, structure, sulfur, long wavelength

## Abstract

Determination of protein crystal structures requires that the phases are derived independently of the observed measurement of diffraction intensities. Many techniques have been developed to obtain phases, including heavy-atom substitution, molecular replacement and substitution during protein expression of the amino acid methionine with selenomethionine. Although the use of selenium-containing methionine has transformed the experimental determination of phases it is not always possible, either because the variant protein cannot be produced or does not crystallize. Phasing of structures by measuring the anomalous diffraction from S atoms could in theory be almost universal since almost all proteins contain methionine or cysteine. Indeed, many structures have been solved by the so-called native sulfur single-wavelength anomalous diffraction (S-SAD) phasing method. However, the anomalous effect is weak at the wavelengths where data are normally recorded (between 1 and 2 Å) and this limits the potential of this method to well diffracting crystals. Longer wavelengths increase the strength of the anomalous signal but at the cost of increasing air absorption and scatter, which degrade the precision of the anomalous measurement, consequently hindering phase determination. A new instrument, the long-wavelength beamline I23 at Diamond Light Source, was designed to work at significantly longer wavelengths compared with standard synchrotron beamlines in order to open up the native S-SAD method to projects of increasing complexity. Here, the first novel structure, that of the oxidase domain involved in the production of the natural product patellamide, solved on this beamline is reported using data collected to a resolution of 3.15 Å at a wavelength of 3.1 Å. The oxidase is an example of a protein that does not crystallize as the selenium variant and for which no suitable homology model for molecular replacement was available. Initial attempts collecting anomalous diffraction data for native sulfur phasing on a standard macromolecular crystallography beamline using a wavelength of 1.77 Å did not yield a structure. The new beamline thus has the potential to facilitate structure determination by native S-SAD phasing for what would previously have been regarded as very challenging cases with modestly diffracting crystals and low sulfur content.

## Abstract 2

The first crystal structure of ThcOx, a cyanobactin oxidase, has been determined to 2.65 Å resolution using S-SAD phasing. This is the first structure reported from the purpose-designed long-wavelength beamline I23 at Diamond Light Source.

## 1. Introduction   

The majority of structures from macromolecular crystals can nowadays be solved by molecular replacement. However, in the absence of suitable homology models, experimental phasing is the method of choice to overcome the crystallo­graphic phase problem. This method requires measurements of the small anomalous differences arising when tuning the wavelength of the X-rays towards the absorption edges of atoms bound to the structures. The most successful label used is selenium by substituting the amino acid methionine by selenomethionine. However, this technique is not universal as it is not compatible with all expression systems and, even if the labelled protein can be produced, crystallization fails in some cases.

The native sulfur single-wavelength anomalous diffraction (S-SAD) method can overcome this by exploiting the intrinsic anomalous signal from sulfur. Over the past years, several studies have demonstrated the applicability of this method to problems of increasing complexity (Liu *et al.*, 2012 ▸; Weinert *et al.*, 2015 ▸; El Omari *et al.*, 2014 ▸; Rose *et al.*, 2015 ▸). On standard experimental setups, typically optimized for wavelengths around the selenium *K* edge (λ = 0.97 Å) and operated in air, S-SAD experiments are performed in the wavelength range 1.7–2.3 Å. The wavelength choice is limited mainly by the increased background noise from air scattering and the reduced signal from air absorption, as well as the size of the detector, given that the diffraction angles needed to measure data at constant resolution increase with the wavelength. To measure the small anomalous signals, high-redundancy data from well diffracting crystals or merging data from multiple isomorphous crystals are needed. The sulfur *K* edge is at λ = 4.96 Å and the anomalous signal increases approximately with the cube of the wavelength as it moves towards the edge. The new long-wavelength MX beamline I23 at Diamond Light Source has been designed to provide an optimized environment for native SAD experiments, enabling experiments at longer wavelengths to increase the anomalous signal with minimal noise (Wagner *et al.*, 2016 ▸).

**Figure 1:**
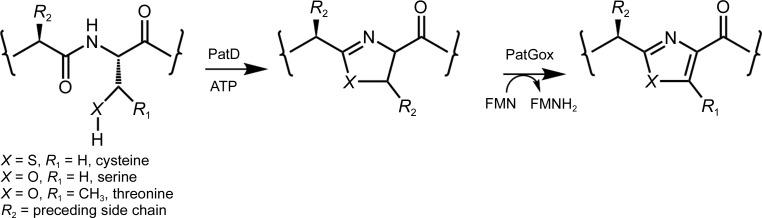
Synthesis schematic of the two-step process to form thiazoles and oxazoles in cyanobactins. The initial step is the ATP-dependent PatD reaction to the form thiazolines and oxazoline, followed by FMN-dependent oxidation by PatGox to yield thiazoles. Note that in related pathways the oxazolines are also oxidized to oxazole rings.

Cyanobactins are a family of ribosomally synthesized and post-translationally modified peptides (RiPPs; Arnison *et al.*, 2013 ▸) from cyanobacteria. Each product is tailored using a selected set of enzymes to produce linear or macrocyclic peptides (Sivonen *et al.*, 2010 ▸). The patellamide pathway (Schmidt *et al.*, 2005 ▸) is the best characterized pathway. The first step in patellamide biosynthesis is the ATP-dependent dehydration of cysteine, threonine and serine by the heterocyclase PatD to form thiazoline, methyl oxazoline and oxazoline, respectively (Fig. 1 ▸). Following this cyclode­hydration, the N-terminus is cleaved off by the PatA protease domain and subsequently macrocyclized by the macrocyclase domain of PatG. Structures have been determined for the heterocyclase (cyclic dehydratase; Koehnke *et al.*, 2013 ▸, 2015 ▸), protease (Houssen *et al.*, 2012 ▸; Agarwal *et al.*, 2012 ▸), prenyltransferase (Bent *et al.*, 2013 ▸), a conserved domain of unknown function (Mann *et al.*, 2014 ▸) and macrocyclase (Koehnke *et al.*, 2012 ▸; Agarwal *et al.*, 2012 ▸) proteins from various cyanobactin pathways. The only enzyme function remaining to be solved in the patellamide cluster is that of the FMN-dependent cyanobactin azoline oxidase. The oxidase converts thiazolines to thiazoles, with some enzymes also capable of the oxidation of oxazolines to oxazoles (Fig. 1 ▸, Table 1 ▸). The oxidase domain is conserved in patellamide-like pathways either as a fused domain to the G protein or as a standalone protein (Martins & Vasconcelos, 2015 ▸); the exception to this is the trunkamide pathway, in which the final compounds are not oxidized.

Here, we present ThcOx, the first novel structure to be solved by in-vacuum long-wavelength macromolecular crystallo­graphy on beamline I23 at Diamond Light Source (Wagner *et al.*, 2016 ▸), and its subsequent high-resolution structure solved by molecular replacement.

## 2. Materials and methods   

### 2.1. Cloning, expression and purification of ThcOx   

The ThcOx gene was amplified from gDNA of cultured *Cyanothece* sp. PCC 7425 obtained from the Pasteur Culture Collection of Cyanobacteria (Paris) and subcloned with an N-terminal *Tobacco etch virus* (TEV) protease-cleavable His_6_ tag followed by a four-glycine linker into a pJexpress 401 vector from DNA2.0 (Table 2 ▸). It was expressed in *Escherichia coli* BL21 (DE3) cells grown on autoinduction medium supplemented with 50 µ*M* riboflavin for 48 h at 20°C. Cell pellets were resuspended in lysis buffer plus EDTA-free protease-inhibitor tablets (Roche) and DNase at 0.4 mg per gram of wet cell pellet. The resuspension was lysed by passage through a cell disruptor at 207 MPa (Constant Systems). The lysate was cleared by centrifugation (40 000*g*, 4°C, 20 min) and then loaded onto an Ni Sepharose 6 Fast Flow column (GE Healthcare) equilibrated with lysis buffer [150 m*M* NaCl, 20 m*M* Tris–HCl pH 8.0, 20 m*M* imidazole pH 8.0, 50 µ*M* flavin mononucleotide (FMN), 3 m*M* β-mercaptoethanol (BME)]. The column was washed with lysis buffer and ThcOx was then eluted with elution buffer (150 m*M* NaCl, 20 m*M* Tris–HCl pH 8.0, 250 m*M* imidazole pH 8.0, 50 µ*M* FMN, 3 m*M* BME). The protein was then passed over a desalting column (16/10 Desalting, GE Healthcare) back into the lysis buffer. TEV protease was added at a mass ratio of 1:10 and the protein was digested for 2 h at 20°C to remove the His_6_ tag. The sample was then loaded onto a second nickel column in lysis buffer and directly onto an ion-exchange column (HiTrap Q Sepharose FF, GE Healthcare) and eluted with a 0.15–1 *M* NaCl gradient. The peak fraction was then concentrated to 7.5 ml (Vivaspin concentrators, 30 kDa molecular-weight cutoff) and applied onto a Superdex 200 gel-filtration column (GE Healthcare) equilibrated with gel-filtration buffer (150 m*M* NaCl, 10 m*M* HEPES pH 7.4, 1 m*M* TCEP). The lack of FMN in the gel-filtration buffer combined with the elution fraction remaining yellow in colour confirmed that FMN was indeed bound to the protein. The protein was concentrated to 4 mg ml^−1^ for crystallography. The purity of the protein was confirmed by SDS–PAGE analysis and its identity was confirmed by mass spectrometry (MS).

### 2.2. Crystallization   

**Figure 2:**
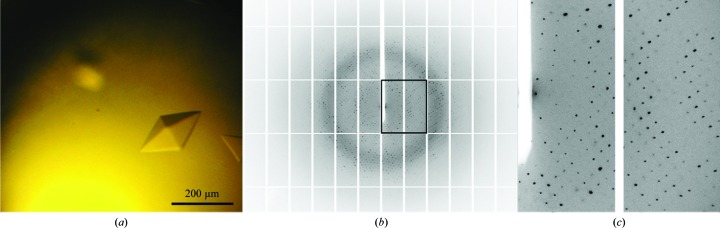
(*a*) Crystals of ThcOx in complex with the peptide substrate NILPQQGQPVIR. (*b*) X-ray diffraction pattern of the high-resolution data collected on beamline I02. (*c*) Enlarged view of the diffraction data.

ThcOx protein was mixed in a 1:1.1 ratio with the peptide NILPQQGQPVIR plus an additional 1 m*M* FMN cofactor and incubated overnight at 273 K prior to setting up crystallization trials. Initial crystallization screening was performed by sitting-drop vapour diffusion in a 96-well Intelli-Plate (300 nl protein mixture plus 150 nl reservoir solution) using a Gryphon robot (Art Robbins; http://www.artrobbins.com/). The only crystals produced from a wide range of screening trials were found in condition No. 16 from Wizard Classic Screen 2 (1.0 *M* sodium citrate, 0.1 *M* CHES pH 9.5) at 293 K. These crystals were subsequently optimized in Linbro hanging-drop plates using a drop volume of 2.5 µl + 1.5 µl over a well of 0.5 ml, with the best crystals found in a condition consisting of 1.0 *M* sodium citrate, 0.1 *M* CHES pH 7.75 at 293 K (Table 3 ▸, Fig. 2 ▸ *a*).

### 2.3. Data collection, processing and phasing   

The best crystals of ThcOx were cryoprotected in paraffin oil and either flash-cooled directly at the synchrotron or cooled and shipped to the synchrotron. The crystals showed a high level of non-isomorphism and diffracted to between 4.0 and 2.65 Å resolution (Figs. 2 ▸ *b* and 2 ▸ *c*). In order to phase the protein structure by molecular replacement, several failed attempts were made using such proteins as putative nitro­reductases from *Anabaena variabilis* (PDB entry 3eo7) and *Ralstonia eutropha* (PDB entry 3hj9), both of which were deposited by the Joint Center for Structural Genomics (JCSG). We were consistently unable to crystallize a selenomethionine variant of the protein under either the native or alternative crystallization conditions. A range of heavy-atom soaks were prepared including sodium bromide, lead(II) acetate, potassium tetrabromoplatinate(II), sodium iodide and potassium iodide, but none of these were successful in obtaining phases. The ThcOx protein contains a total of ten S atoms after removal of the His_6_ tag (Table 2 ▸). With a total of 477 amino-acid residues, the Bijvoet ratio at a wavelength of 2.0 Å is 0.98%. Our attempts to solve the structure by S-SAD phasing with in-house data collected at a wavelength of 1.54 Å or on beamline I02 at Diamond Light Source using data collected at a wavelength of 1.77 Å both failed owing to the low anomalous signal at those wavelengths.

**Figure 3:**
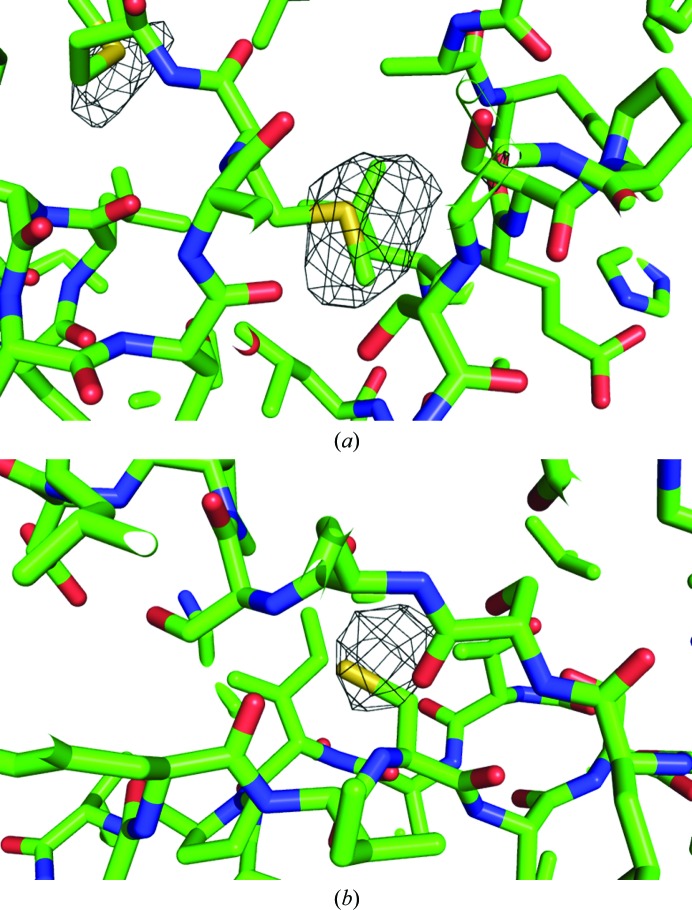
The phased anomalous difference (*F* ^+^ − *F* ^−^) map shows the accuracy of the anomalous difference measurements collected on beamline I23 using a wavelength of 3.1 Å. The map is contoured around the sulfur-containing residues (*a*) methionine and (*b*) cysteine at 3.5σ. The phases were calculated from a molecular-replacement solution using the final (high-resolution) structure as the search model. (Image from *PyMOL*.)

We therefore used the new long-wavelength in-vacuum beamline I23 with its custom detector to investigate the use of longer wavelengths for phasing. A wavelength of 3.1 Å appeared to be a good compromise between the increase in the anomalous signal and the decrease in data quality owing to the greater sample absorption at longer wavelengths. At this wavelength the Bijvoet ratio is 2.1%, which is more than 2.5 times the value at a wavelength of 1.77 Å. Owing to the large crystal-to-crystal variability, 48 crystals had to be transferred into the beamline vacuum end station before a crystal of suitable quality could be found. The best crystal diffracted to a resolution limit of 3.15 Å. Data were collected with the large in-vacuum PILATUS 12M detector (Dectris, Switzerland) at a wavelength of 3.1 Å with 0.1° oscillations and 0.1 s exposure time. A total of 4000 images were measured in inverse-beam mode with wedge sizes of 20° and were subsequently processed and scaled with *XDS*/*XSCALE* (Kabsch, 2010 ▸). No additional absorption correction was performed. The substructure was determined with *SHELXD* (Sheldrick, 2010 ▸) with a success rate of one hit in 1000 trials using a resolution cutoff of 4.2 Å when searching for 18 sites. Using this substructure, *AutoSol* (Terwilliger *et al.*, 2009 ▸) found non­crystallographic symmetry, and after density modification automatic model building placed about 20% of the amino-acid residues in an electron-density map of sufficient quality to allow manual model building. The accuracy of the anomalous measurements can be seen in a phased anomalous difference map, which shows strong peaks for the S atoms (Fig. 3 ▸).

This initial model was refined and then used as a search model for molecular replacement to solve a previous data set of higher resolution collected on beamline I02 at Diamond Light Source. The high-resolution data were processed and scaled in *xia*2 (Winter, 2010 ▸) using *XDS* (Kabsch, 2010 ▸) and *SCALA* (Evans, 2006 ▸). The structure was determined using *Phaser* (McCoy *et al.*, 2007 ▸) as part of the *CCP*4 suite (Winn *et al.*, 2011 ▸). All data-collection statistics can be found in Table 4 ▸.

### 2.4. Refinement   

The high-resolution model was refined by iterative cycles of manual rebuilding using *Coot* (Emsley *et al.*, 2010 ▸) and refinement using *REFMAC*5 (Murshudov *et al.*, 2011 ▸) in the *CCP*4 suite (Winn *et al.*, 2011 ▸). TLS restraints were calculated using the *TLSMD* server (Painter & Merritt, 2006 ▸) and were used in refinement (Winn *et al.*, 2001 ▸). Final refinement statistics can be found in Table 5 ▸. The structure was validated using *MolProbity* (Chen *et al.*, 2010 ▸) and the coordinates were deposited in the Protein Data Bank (PDB entry 5lq4). The anomalous data have also been deposited.

## 3. Results   

**Figure 4:**
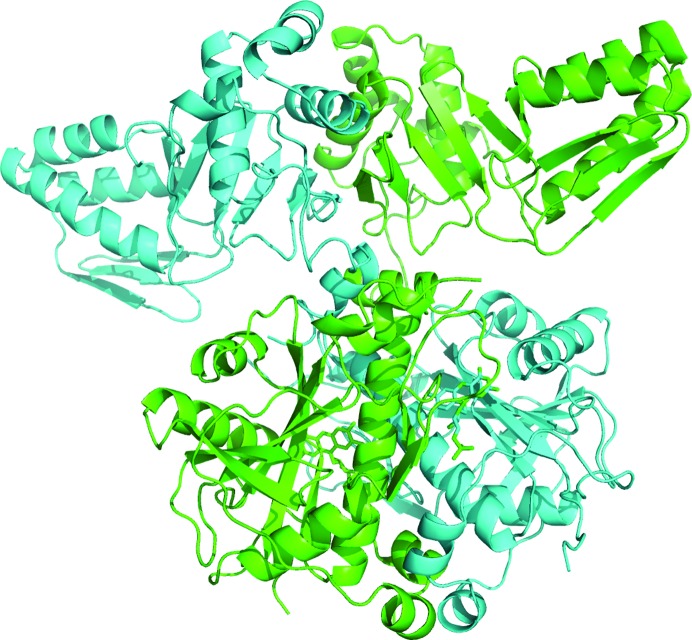
Cartoon representation of ThcOx highlighting the two molecules (green and cyan) in the asymmetric unit. (Image from *PyMOL*.)

The protein crystals belonged to space group *P*4_1_2_1_2_1_, with two molecules found in the asymmetric unit. Simple phase extension from the long-wavelength data used for phasing was not possible owing to non-isomorphism between the two crystals. The *c* axis is more than 10% larger in the long-wavelength data, and the unit-cell volume is about 15% larger compared with the data obtained from the crystal which diffracted to the highest resolution. Hence, molecular replacement was needed to phase the high-resolution data. This crystal-to-crystal variability was typical. The structure is composed of a novel domain and an FMN nitroreductase domain. Each molecule in the asymmetric unit consists of 16 α-helices and 17 β-sheets (Fig. 4 ▸; structural figures were created using *PyMOL* v.1.5.0.4 from Schrödinger). The final structure contains residues 3–222 and 233–469 in chain *A* and 1–222 and 231–473 in chain *B*. In both monomers, the missing residues are located between α-helices 7 and 8 (the linker between the two domains) and at the N- and C-termini. These residues are presumed to be disordered in the structure. There are four cysteine residues per molecule, none of which form disulfide bonds.

*PISA* (Krissinel & Henrick, 2007 ▸; Krissinel, 2010 ▸) analysis infers that the dimer is biologically relevant, with each molecule containing a single FMN. The N-terminal domain contains a portion (residues 7–86) which, by visual analysis, supported by *Phyre*2 (Kelley & Sternberg, 2009 ▸), possesses the same fold as the leader binding domain of TruD (Koehnke *et al.*, 2013 ▸), the so-called ‘peptide-clamp domain’. These domains are common in the RiPP family (Melby *et al.*, 2014 ▸), and its orientation here suggests a possible role in function. The remaining residues in domain 1 (87–194) form a second peptide-clamp domain, but its binding site is buried by an interaction with the other clamp domain and thus it is unlikely to be active.

**Figure 5:**
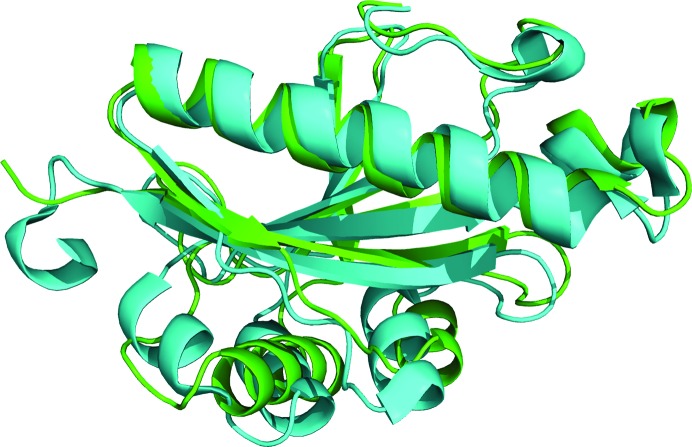
Structural alignment of the second domain of ThcOx (residues 323–469) and an FMN nitroreductase (PDB entry 3eo7; residues 97–245), showing a high degree of homology (r.m.s.d. = 1.452 Å). (Image from *PyMOL*.)

The C-terminal domain (residues 323–469) contains the FMN molecule and has a high degree of homology (r.m.s.d. = 1.45 Å) to the putative nitroreductase from *A. variabilis* (PDB entry 3eo7; residues 97–245; Fig. 5 ▸). Crystals were only observed when co-crystallized in the presence of the peptide NILPQQGQPVIR. The peptide was included as there was evidence that it may bind to the oxidase; however, there was no evidence of any density for the peptide. We have attempted to achieve a complex structure with substrate bound at the catalytic site but have so far been unsuccessful.

## 4. Discussion   

We have reported the first structure of an oxidase protein from a cyanobactin pathway. The structure was determined by native S-SAD phasing on the new I23 beamline at Diamond Light Source. A wavelength of 3.10 Å was used to measure a sufficiently large anomalous signal to experimentally phase the structure of ThcOx from a crystal which diffracted to 3.15 Å resolution with useful anomalous data extending to only 4.2 Å resolution. These resolution limits would traditionally be unfavourable for phase determination by native S-SAD, particularly given the large size of the asymmetric unit (a dimer of 954 residues in total) with relatively few S atoms. Furthermore, the inability to extend the phases to a higher resolution data set, caused by non-isomorphism, required that the initial phases at 3.15 Å resolution had to be of sufficient quality to allow model building. Thus, ThcOx represented a particularly challenging test for the I23 beamline and the successful structure determination validates its performance.

This structure determination demonstrates the great potential of the novel in-vacuum long-wavelength MX beamline I23. The combination of the in-vacuum sample environment with the large semi-cylindrical PILATUS 12M pixel hybrid detector allows very accurate measurements with minimal background, giving very high signal-to-noise ratios in a wavelength range typically not accessible at standard MX beamlines. This new beamline should significantly extend the range of protein crystals that are amenable to native S-SAD structure determination.

## Supplementary Material

PDB reference: ThcOx, 5lq4

## Figures and Tables

**Table 1:** PatGox and homologues

Enzyme	Fused/standalone	Natural products contain	Organism/biosynthetic pathway
PatGox	Fused, part of PatG	Thiazoles, oxazolines	*Prochloron* sp./patellamides
ThcOx	Standalone	Thiazoles, thiazolines, oxazolines	*Cyanothece* PCC 7425/cyanothecamides
ThcOx2	Standalone	Thiazoles, thiazolines, oxazolines	*Cyanothece* PCC 7822
TriOx	Standalone	Thiazoles	*Trichodesmium erythraeum* ISM101/trichamide
McaGox	Fused, part of McaG	Thiazoles, oxazoles	*Microcystis aeruginosa* NIES-298/microcyclamide
ArtGox	Fused, part of ArtG	Thiaozles	*Arthrospira spirulina*/arthrospiramides
LynGox	Fused, part of LynG	Thiazoles	*Lyngbya aestuarii* CCY9616/aestuaramides
TenGox	Fused, part of TenG	Thiazoles, oxazoles	*Nostoc spongiaeforme* var. *tenue*/tenuecyclamide

**Table 2:** Macromolecule-production information for ThcOx from *Cyanothece* sp. PCC 7425

Source organism	*Cyanothece* sp. PCC 7425 obtained from the Pasteur Culture Collection of Cyanobacteria (Paris)
DNA source	gDNA extracted from cultured *Cyanothece* sp. PCC 7425 on BG-11 medium with 12 h light/12 h dark cycles. Subcultures on fresh media were prepared after four weeks incubation.
Expression vector	pJexpress 401 (DNA2.0)
Expression host	*E. coli* BL21 (DE3)
Complete amino-acid sequence of the construct produced†	MHHHHHHENLYFQ\GGGGMLDLFTLSFSPDLSIASEAEQLTLQSKDDRLILEHPQPGLRTALEQLKQGNLTLAQLTELVSEQDGVEAGITFASELEKLVDLGWICHSVLPLITAIPIAKDYELNVPDSSWQTTAIALSRFAFLHQDLQQLVLESPRSKSKLVILDWRVGAVIAKLAQSDRGFIFATSADSLLADLSLELEELKRLFALLIATQMMDLEPEDETITQWKFHNLLFHHYTRLGRLDNSRKLNLPVFEHRDRYPYVKPVISTQAIPLVKPDLTALATTDMTLTEAIETRRSIREYSDQPITLAQLGEFLYRCARVKAVYTLPEDPMQVGESTTRPYPSGGALYELEIYPLVHQCGDLAAGLYHYQPLSHTLHPVADWTPEVESLVYDAWRATGQQSIPQIVLIITARFGRLFWKYHDIAYSLILKHVGVLYQTFYLVATAMQLAPSAIGAGNTTKFCQIAGLNPDEEASVGEFSLGAAKPQQQS

† The *Tobacco etch virus* (TEV) protease cleavage point is marked \.

**Table 3:** Crystallization

Method	Hanging-drop vapour diffusion
Plate type	Linbro 24-well
Temperature (K)	293
Protein concentration	4 mg ml^−1^ plus peptide (1:1.1) (pre-loaded with FMN)
Buffer composition of protein solution	150 m*M* NaCl, 10 m*M* HEPES pH 7.4, 1 m*M* TCEP
Composition of reservoir solution	1.0 *M* sodium citrate, 0.1 *M* CHES pH 7.75
Volume and ratio of drop	2.5 µl + 1.5 µl
Volume of reservoir (ml)	0.5

**Table 4:** Data collection and processing Values in parentheses are for the outer shell.

Diffraction source	Diamond Light Source beamline I23	Diamond Light Source beamline I02
Wavelength (Å)	3.096	1.771
Temperature (K)	∼50†	100
Detector	PILATUS 12M	PILATUS 6M
Space group	*P*4_1_2_1_2	*P*4_1_2_1_2
*a*, *b*, *c* (Å)	111.4, 111.4, 217.6	109.3, 109.3, 195.4
α, β, γ (°)	90, 90, 90	90, 90, 90
Resolution range (Å)	217.55–3.15 (3.37–3.15)	72.84–2.65 (2.72–2.65)
Total No. of reflections	575020 (86233)	285753 (21726)
No. of unique reflections	22295 (3452)	35204 (2542)
Completeness (%)	91.6 (80.6)	100.0 (99.6)
Anomalous completeness (%)	92.1 (81.4)	100.0 (99.6)
Multiplicity	25.8 (25.0)	8.1 (8.5)
〈*I*/σ(*I*)〉	28.8 (2.3)	17.9 (2.7)
*R*_meas_	0.077 (1.617)	0.089 (0.890)
Overall *B* factor from Wilson plot (Å^2^)	121	60

† An accurate temperature calibration is still outstanding; this value is based on a temperature of 44 K at the goniometer head, assuming a temperature rise of around 6 K through the sample mount.

**Table 5:** Structure solution and refinement Values in parentheses are for the outer shell.

Resolution range (Å)	72.83–2.65
Completeness (%)	99.9
No. of reflections, working set	33383
No. of reflections, test set	1751
Final *R*_cryst_	0.197
Final *R*_free_	0.228
No. of non-H atoms	
Protein	7289
Ligand	62
Water	4
R.m.s. deviations	
Bonds (Å)	0.008
Angles (°)	1.29
Average *B* factors (Å^2^)	
Protein	76
Ligand (FMN)	48
Water	63
Ramachandran plot	
Most favoured (%)	94.5
Allowed (%)	99.1
